# Association of the metabolic score for insulin resistance with cardiovascular diseases, cardiovascular and all-cause mortality in Chinese hypertensive population

**DOI:** 10.3389/fendo.2023.1326436

**Published:** 2024-03-08

**Authors:** Liting Zhang, Chao Yu, Tao Wang, Wei Zhou, Huihui Bao, Xiaoshu Cheng

**Affiliations:** ^1^ Department of Cardiovascular Medicine, the Second Affiliated Hospital of Nanchang University, Nanchang of Jiangxi, China; ^2^ Center for Prevention and Treatment of Cardiovascular Diseases, the Second Affiliated Hospital of Nanchang University, Nanchang of Jiangxi, China; ^3^ Jiangxi Provincial Cardiovascular Disease Clinical Medical Research Center, Nanchang of Jiangxi, China; ^4^ Jiangxi Sub-center of National Clinical Research Center for Cardiovascular Diseases, Nanchang of Jiangxi, China

**Keywords:** insulin resistance, METS-IR, cardiovascular events, cardiac death, all-cause mortality, shock

## Abstract

**Importance:**

Little is known about the relationship between the metabolic score for insulin resistance (METS-IR) and the prognosis of hypertensive patients in China.

**Objective:**

To investigate the association between the novel non–insulin‐based METS-IR index and the cardiovascular composite endpoints and all-cause mortality in Chinese hypertensive participants.

**Design, setting, and participants:**

This cohort study used data from the China H-Type Hypertension Project, a long-term prospective cohort consisting of 14234 hypertensive patients in southern China, with a baseline from March to August 2018. The median follow-up period for participants was 3.94 years, as of 2022. The data analysis period is from July 2023 to September 2023.

**Exposures:**

METS-IR index of participants in the Chinese H-type hypertension project. The calculation formula for METS-IR is (Ln (2 × FPG) +TG) × BMI/Ln (HDL-C).

**Main outcomes and measures:**

Cardiovascular events and cardiovascular, all-cause mortality were identified by linking the cohort database with the health care system through October, 2023.

**Results:**

A total of 14220 participants were included in this study. The prevalence rates of cardiovascular disease (CVD), cardiovascular death, and all-cause death were 2.59% (369/14220), 2.79% (397/14220), and 5.66% (805/14220), respectively. After adjusting for confounding factors in the multivariate logistic regression analysis models, the METS-IR index was significantly positively correlated with CVD, and cardiovascular, all-cause mortality, whether as a categorical or continuous variable. Layered analysis showed that the METS-IR index of hypertensive participants in different subgroups was positively correlated with the endpoint event.

**Conclusions and relevance:**

This large, prospective cohort study demonstrated that the METS-IR index, a new IR evaluation index, were independently associated with a higher risk of the cardiovascular composite endpoint and all-cause mortality among Chinese hypertensive population. Importantly, our finding provides an independent indicator for evaluating the prognosis of hypertensive patients.

## Introduction

Hypertension (HTN) is now widely recognized as a threat to global development and one of the causes of death and disability in the global population. According to China Cardiovascular Health and Disease Report 2019, the number of hypertensive patients in China has reached 245 million until 2019 (1). According to some data, the incidence of insulin resistance in HTN patients is 58% (2). From these, it can be seen that abnormal glucose and lipid metabolism in patients with HTN is very common and significantly increases the risk of cardiovascular diseases (CVD), including severe complications of hypertension such as stroke, coronary heart disease, and heart failure and so on. The poor prognosis of hypertensive patients can cause a heavy burden on their families and society. Therefore, seeking better predictive indicators for glucose and lipid metabolism abnormalities in HTN patients may help alleviate the huge burden of global healthcare expenditure (3).

Insulin resistance (IR), a state of systemic insulin sensitivity decline in the body, is a key mechanism of glucose and lipid metabolism disorders. According to current epidemiological and pathophysiological studies, IR may be a potential major cause of CVD (4–6). The gold standard for evaluating IR is the hyperinsulinemic normal blood glucose clamp (HEC), which is not suitable for large-scale epidemiological investigations due to its complicated and expensive procedures. The METS-IR index is a newly developed index that includes laboratory indicators such as fasting blood glucose (FBG), triglycerides (TG), high-density lipoprotein (HDL-C), and body mass index. It aims to become a practical and effective alternative biomarker for IR (7). Compared with other non–insulin‐based IR indices such as TG/HDL and TyG index (triglyceride glucose index), the METS-IR index has a stronger correlation with HEC (7).

Previous studies have reported on the relationship between TyG index and cardiovascular events and mortality, as well as the relationship between TG/HDL index and CVDs and mortality (8–16). In these literatures, there are not only cross-sectional studies but also prospective studies, with both positive and negative relationships. It is worth noting that, to our knowledge, studies describing the relationship between METS-IR index and CVD are mainly cross-sectional studies (3, 17–20). There are few longitudinal studies on this topic, and they are limited in the general population, reporting the association between METS-IR and CVD risk (17, 19). Few studies have further investigated the relationship between METS-IR and CVD, cardiovascular and all-cause mortality, especially in patients with hypertension. Therefore, the purpose of this article is to investigate the relationship between the METS-IR index and cardiovascular composite endpoints and all-cause mortality in Chinese hypertensive patients.

## Methods

This study was conducted in accordance with Helsinki Declaration and approved by the Ethics Committee of the Biomedical Research Institute of Anhui Medical University. All participants sign a written informed consent form. This study follows the reporting guidelines of the Strengthening Epidemiological Observation Research Report (STROBE).

### Study population

The registration study of H-type hypertension in China is a continuous dynamic cohort study (registration number: ChiCTR1800017274). If the systolic blood pressure ≥ 140mmHg and/or the diastolic blood pressure ≥ 90mmHg, and homocysteine (Hcy) level ≥ 10mmol/L, it is defined as H-type hypertension. The main purpose of this study is to establish a registry of Chinese patients with H-type hypertension, investigate the prevalence and treatment rates of hypertension in China, and evaluate relevant factors affecting hypertension and its prognosis (21). From March 2018 to August 2018, our research team recruited a total of 14234 hypertensive participants in Wuyuan County, Jiangxi Province, China, and conducted a baseline survey, including face-to-face interviews, physical examinations, and blood biochemical indicators testing and so on. In June 2022, we returned to Wuyuan County, Jiangxi Province and followed up 14232 (99.9%) hypertensive patients for the first time. In 2019, due to the impact of the novel coronavirus pandemic, which led to the reduction of population mobility and the special concern of personal information by the Chinese government, the follow-up rate of this study was so high. In short, this study was a multicenter observational study conducted in Wuyuan City, Jiangxi Province, China in March 2018, with a median follow-up of 3.94 years.

The inclusion criteria for this study are adults aged 18 years and above with HTN, defined as 1) blood pressure measured after 5 minutes of sitting, with resting systolic blood pressure (SBP) ≥ 140 mmHg and/or diastolic blood pressure (DBP) ≥ 90 mmHg; 2) Take antihypertensive drugs during the screening period; 3) participants who have been previously diagnosed with hypertension. The exclusion criteria are 1) the patient has a history of mental illness or cannot cooperate with the staff in this study; 2) Patients are unable to follow up or plan short-term relocation according to the research protocol; 3) Patients assessed by the research physicians as unsuitable for inclusion or long-term follow-up.

Therefore, a total of 14234 participants completed this study, excluding patients with missing death information (n=2), patients with missing blood biochemical tests (n=7), and patients with missing BMI values (n=5). Finally, a total of 14220 patients were included in the final study. The patient screening process diagram is shown in **Supplementary Figure 1**.

### Baseline characteristics

The demographic characteristics (such as age and gender), lifestyle (such as smoking and drinking), medical history (such as diabetes, family history of coronary heart disease, family history of hypertension) and drug use (such as antihypertensive drugs, hypoglycemic drugs and lipid-lowering drugs) of participants were collected by trained researchers through questionnaires. They also collected anthropometric indicators of participants, including weight, height, blood pressure, and so on.

After participants fasted overnight for at least 12 hours, experienced researchers collected blood samples from them. At the core laboratory of the National Kidney Disease Clinical Research Center in Guangzhou, China, an automated clinical analyzer (Beckman Colter) was used to measure laboratory values of FBG, low-density lipoprotein (LDL-C), HDL-C, and TG. To be precise, METS-IR uses the formula (Ln (2 × FPG (mg/dL)) + TG (mg/dL)) × BMI (kg/m2)/Ln (HDL-C(mg/dL)) calculation. All laboratory measurements comply with standardization and certification procedures.

### Ascertainment of mortality

The life statuses of participants in this study are preliminarily confirmed through the Chinese cause of death registration system. Subsequently, well-trained staffs will conduct telephone follow-up to supplement and further review through the Chinese medical system. The endpoint event committee, composed of experts, will ultimately determine the endpoint events. The outcome variables of this study include cardiovascular composite endpoints and all-cause mortality. The cardiovascular composite endpoint includes CVDs and cardiovascular mortality. CVDs are defined as first myocardial infarction, stroke, hospitalization for unstable angina, arterial revascularization surgery, or cerebrovascular disease or cardiovascular death. Among them, the classification of cardiovascular mortality is mainly based on the International Classification of Diseases 10th Revision (ICD-10) code, and the causes of death include heart disease (I00-109, I11, I13, I20-151) or cerebrovascular disease.

### Statistical analysis

The data is represented by the mean ± standard deviation (SD) of continuous variables and the frequency (%) of categorical variables. Patients were divided into four groups based on the quartiles of the METS-IR index or survival status, and baseline characteristics were compared between different groups using analysis of variance or chi square tests (when the variables satisfy a normal distribution), Kruskal Wallis analysis (when the variables do not satisfy a normal distribution). Continuous variables use the median to estimate the missing values of covariates (<4.4%), while categorical variables use missing indicators to estimate the missing values of covariates.

The Cox proportional risk regression model (risk ratio [HR] and 95% CI) was used to examine the association between the METS-IR index and cardiovascular composite endpoints and all-cause mortality in hypertensive patients. In Model 1, we adjusted for age (continuous) and gender (male and female), BMI (continuous). In Model 2, we further adjusted systolic blood pressure (continuous), diastolic blood pressure (continuous), AST (continuous), ALT (continuous), antihypertensive drugs (yes or no), lipid-lowering drugs (yes or no), hypoglycemic drugs (yes or no), and current smoking status (yes or no). Moreover, smoothing curves (restricted cubic spline method) were graphed to visually evaluate the effects of METS-IR index levels on outcome events.

What’s more, to observe whether METS-IR has any additional predictive value compared to established clinical risk variables, we attempted to fit it into a logistic regression model and compare using the C-statistic, continuous net reclassification improvement (NRI), and comprehensive discriminant improvement (IDI).

In addition, further stratified analysis was conducted based on different cardiovascular risk factors, age (<60 or ≥ 60 years old), gender (female or male), BMI (<18.5 kg/cm2, 18.5-24 kg/cm2 or ≥ 24 kg/cm2), and current smoking (yes or no) and alcohol consumption (yes or no), to determine the consistency of the impact of the METS-IR index on the endpoints. The robustness of the relationship between the METS-IR index and the endpoint was further tested. We also conducted several sensitivity analyses. Firstly, all participants were analyzed and the METS-IR index was included as a categorical variable and a continuous variable in the model analysis. Secondly, participants were included in models based on gender differences. Thirdly, incorporate the METS-IR index using different grouping methods into the model. Fourthly, we conducted a competing risk approach (i.e. Fine-Gray models) analysis between METS-IR and cardiovascular mortality, with non-cardiovascular death handled as a competing outcome. Finally, we excluded participants who used hypoglycemic and lipid-lowering drugs before baseline to minimize potential reverse causal bias.

All statistical analyses were conducted using Empower (R; www.empowerstats.com; X&Y Solutions, Inc, Boston, MA, USA) and statistical software package (R) (http://www.R-project.org R Foundation). All P-values are double tailed, and P<0.05 is considered statistically significant.

## Results

### Characteristics of participants

According to the quartiles grouping of METS-IR index, the baseline characteristics of participants in this study are shown in **Table 1** (mean [SD] age, 63.8 [9.3] years old; 6714 [47.2%] males and 7506 [52.8%] females). Compared with participants in the Q1 group of the METS-IR index, participants in the Q4 group had higher height (156.9 [8.3] cm), heavier weight (68.1 [9.5] kg), lower average systolic blood pressure (147.7 [17.3] mmHg vs 149.3 [19.1] mmHg), and higher average diastolic blood pressure (91.4 [10.6] mmHg vs 86.4 [11.2] mmHg). In addition, patients with higher METS-IR index have higher levels of triglycerides, LDL, FBG, AST, and ALT, while HDL levels are lower.

**Table 1:** Baseline characteristics of the study population according to METS-IR index quartiles.

Variables	Total	Q1(n=3555)	Q2(n=3555)	Q3(n=3555)	Q4(n=3555)	P value
Age, mean ± SD, year	63.8 ± 9.4	67.9 ± 8.9	64.7 ± 8.8	62.6 ± 8.8	60.1 ± 9.2	<0.001
sex, n (%)						<0.001
Male	6714(47.2%)	1896 (28.2%)	1609 (24.0%)	1557 (23.2%)	1652 (24.6%)	
Female	7506(52.8%)	1659 (22.1%)	1946 (25.9%)	1998 (26.6%)	1903 (25.4%)	
Height, cm	156.1 ± 8.2	155.5 ± 8.1	155.6 ± 8.1	156.3 ± 8.2	156.9 ± 8.3	<0.001
Weight, cm	57.7 ± 10.7	47.5 ± 6.5	54.7 ± 6.7	60.4 ± 7.4	68.1 ± 9.5	<0.001
BMI, kg/m2	23.6 ± 3.7	19.6 ± 1.7	22.5 ± 1.5	24.7 ± 1.7	27.7 ± 3.6	<0.001
Mean SBP, mmHg	148.4 ± 17.9	149.3 ± 19.1	148.2 ± 17.3	148.3 ± 17.7	147.7 ± 17.3	<0.001
Mean DBP, mmHg	88.9 ± 10.8	86.4 ± 11.2	88.2 ± 10.3	89.7 ± 10.4	91.4 ± 10.1	<0.001
HR, bmp	76.7 ± 14.2	75.7 ± 15.2	75.9 ± 14.2	76.6 ± 13.5	78.40 ± 13.7	<0.001
Current smoking	3658(25.7%)	1195 (32.7%)	886 (24.2%)	772 (21.1%)	805 (22.0%)	<0.001
Current drinking	3062(21.5%)	925 (30.2%)	770 (25.1%)	696 (22.7%)	671 (21.9%)	<0.001
Use of hypoglycemic drugs	754(5.3%)	47(6.2%)	134(17.8%)	220(29.2%)	353(46.8%)	<0.001
Use of lipid-lowering drugs	506(3.6%)	66(13.0%)	106(20.9%)	144(28.9%)	190(37.5%)	<0.001
Use of antihypertensive drugs	9219(64.8%)	2152(23.3%)	2132(23.1%)	2355(25.5%)	2400(26.0%)	<0.001
Education						<0.001
Primary and below	9207(79.9%)	2425 (86.6%)	2296(82.1%)	2274(78.4%)	2212(73.2%)	
Secondary school	2135(18.5%)	359(12.8%)	456(16.3%)	579(20.0%)	741(24.5%)	
University and above	176(1.5%)	17 (0.6%)	44 (1.6%)	47 (1.6%)	68 (2.3%)	
Life level						<0.001
Rich	1503(13.0%)	339(12.1%)	360(12.9%)	379(13.1%)	425(14.1%)	
Ordinary	7741(67.2%)	1832(65.4%)	1874(67.0%)	2005(69.1%)	2030(67.2%)	
Poor	2274(19.7%)	630(22.5%)	562(20.1%)	516(17.8%)	566(18.7%)	
Laboratory indicators						
FBG, mmol/L	6.2 ± 1.6	5.8 ± 0.9	6.0 ± 1.2	6.2 ± 1.5	6.8 ± 2.3	<0.001
TG, mmol/L	1.8 ± 1.3	1.1 ± 0.5	1.5 ± 0.7	1.9 ± 0.9	2.8 ± 1.8	<0.001
HDL, mmol/L	1.6 ± 0.4	1.9 ± 0.4	1.6 ± 0.4	1.5 ± 0.3	1.3 ± 0.3	<0.001
LDL, mmol/L	3.0 ± 0.8	2.8 ± 0.8	3.0 ± 0.8	3.1 ± 0.8	3.01± 0.8	<0.001
AST, mmol/L	26.8 ± 15.8	27.6 ± 15.4	25.7 ± 12.1	26.0 ± 10.5	27.9 ± 22.5	<0.001
ALT, mmol/L	20.5 ± 16.6	16.8 ± 11.1	18.2 ± 13.2	21.0 ± 13.4	26.1 ± 24.0	<0.001
METS-IR	34.6 ± 7.2	26.2 ± 2.4	31.8 ± 1.3	36.4 ± 1.4	43.9 ± 5.4	<0.001

BMI, body mass index; SBP, systolic blood pressure; DBP, diastolic blood pressure; HR, heart rate; FBG, fasting blood glucose; TG, triglyceride; HDL, high-density lipoprotein; LDL, low-density lipoprotein; METS-IR, metabolic score for insulin resistance; AST, aspartate transaminase; ALT, alanine transaminase.

### Association of METS-IR with cardiovascular endpoints and mortality

**Table 2** shows the relationship between METS-IR index and cardiovascular composite endpoints, as well as the risk of all-cause mortality. When Mets-IR index was checked as a continuous variable, in a fully adjusted model (model 2), it was found that for every additional SD in Mets-IR index, the risk of developing CVD increased by 66% (HR=1.66; 95% CI, 1.30-2.12); the risk of cardiovascular death increases by 57% (HR=1.57; 95% CI, 1.22-2.02); the risk of all-cause death increases by 33% (HR=1.33; 95% CI, 1.11-1.60). When METS-IR index is checked as a categorical variable, in Model 1, the risks of developing CVD are 61% (95% CI 1.16-2.22), 112% (95% CI 1.41-3.20), and 234% (95%CI 1.98-5.65) for the Q2, Q3, and Q4 groups, respectively, compared to the Q1 group of Mets-IR index. In model 2, similar results can be observed. The differences are that in Model 1, compared to Q1 of Mets-IR index, the Q3 and Q4 quartiles are associated with a higher risk of all-cause mortality (HR=1.33, 95%CI=1.01-1.76, HR=1.64, 95%CI=1.13-2.37); However, no such increase risk was observed in Q2 group (HR=1.12, 95%CI=0.90-1.38). In Model 2, only the Q4 group could observe an increase risk (HR=1.53, 95%CI=1.05-2.23). The results of cardiovascular mortality are similar to the above. When the METS-IR index categorical variable is treated as a continuous variable, the statistical significance (p for trend<0.05) of METS-IR index trends in different models is consistent.

**Table 2:** Multivariable-adjust HRs and 95%CI of the METS-IR index quartiles associated with CVD, Cardiovascular Death, All-cause Death.

Variables	Event, n (%)	Model 1		Model2	
		HR (95%CI)	P Value	HR (95%CI)	P Value
CVD					
METS-IR Per SD		1.78(1.41,2.25)	<0.001	1.66(1.30-2.12)	0.001
METS-IR Quartiles					
Q1	120(3.38%)	1 [Reference]		1 [Reference]	
Q2	97(2.78%)	1.61 (1.16, 2.22)	0.004	1.51(1.09,2.10)	0.013
Q3	80(2.25%)	2.12 (1.41, 3.20)	<0.001	1.90(1.26,2.89)	0.002
Q4	72(2.03%)	3.34 (1.98, 5.65)	<0.001	2.89(1.69,4.94)	<0.001
P For Trend			<0.001		<0.001
Cardiovascular Death					
METS-IR Per SD		1.58(1.23,2.02)	<0.001	1.57(1.22,2.02)	<0.001
METS-IR Quartiles					
Q1	162(4.56%)	1 [Reference]		1 [Reference]	
Q2	101(2.84%)	1.29(0.95,1.75)	0.107	1.28(0.94,1.75)	0.111
Q3	80(2.25%)	1.59(1.07,2.37)	0.021	1.56(1.04,2.33)	0.030
Q4	54(1.52%)	1.86(1.10,3.17)	0.021	1.79(1.04,3.08)	0.034
P For Trend			0.010		0.024
All-cause Death					
METS-IR Per SD		1.36(1.14,1.63)	0.001	1.33(1.11,1.60)	<0.001
METS-IR Quartiles					
Q1	349(9.82%)	1 [Reference]		1 [Reference]	
Q2	195(5.49%)	1.12(0.9,1.38)	0.316	1.10(0.88,1.36)	0.399
Q3	151(4.25%)	1.33(1.01,1.76)	0.045	1.28(0.96,1.69)	0.091
Q4	110(3.09%)	1.64(1.13,2.37)	0.009	1.53(1.05,2.23)	0.026
P For Trend			0.007		0.023

Model 1 adjusted for age, sex, BMI.

Model 2 adjusted for age, sex, BMI, current smoking, mean systolic blood pressure; mean diastolic blood pressure, hypoglycemic drugs, hypoglycemic drugs, lipid-lowering drugs.

METS-IR, metabolic score for insulin resistance; CVD, cardiovascular disease; BMI, body mass index.

**Figure 1:**
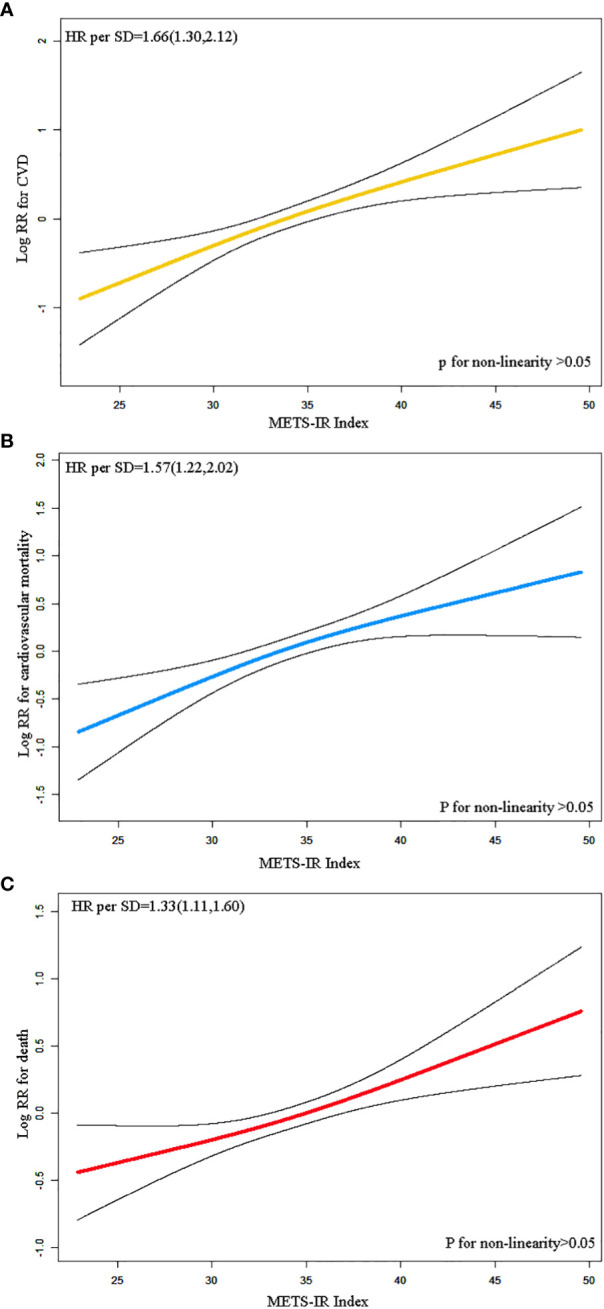
Restricted cubic spline regression analysis of METS-IR index with CVD **(A)**, cardiovascular death **(B)**, all-cause death **(C)**.

**Figure 1** illustrates the dose-response relationship between Mets-IR index and the risk of CVD in **Figure 1A** (yellow), cardiovascular death in **Figure 1B** (blue), and all-cause death in **Figure 1C** (red). These results indicate that there is a linear dose-response relationship between Mets-IR index and the risk of CVD, cardiovascular death, and all-cause death (P overall<0.05, P non- linear>0.05).

### Additive predictive value of METS- IR

It could be seen that the effect of continuous NRI and IDI on predicting cardiovascular mortality was significantly improved after adding Mets-IR to the basic model, with a respective increase of 0.091 (P=0.047) and 0.002 (P=0.027). Furthermore, the additional ability of METS-IR to predict CVD and all-cause mortality had also been confirmed, with an increase of 0.128(P=0.027) and 0.001(P=0.004), respectively (**Supplementary Table 5**).

### Subgroup analysis

**Figure 2:**
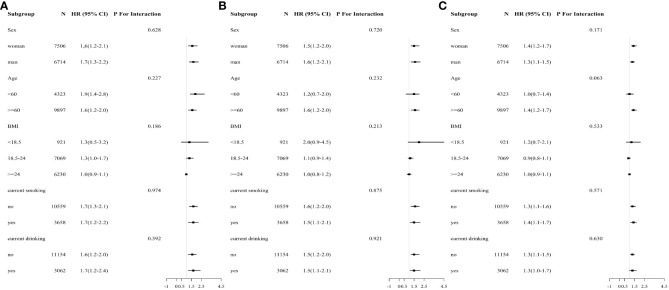
Subgroup and interaction analysis between METS-IR (Per SD) and **(A)** CVD, **(B)** cardiovascular mortality, **(C)** all-cause mortality across various subgroups.

The correlation between METS-IR index (per SD increment) and the risk of CVD, cardiovascular death, and all-cause death was evaluated through stratified analysis based on age, gender, BMI, current smoking status, and current alcohol consumption (**Figure 2**). No interaction was observed between the selected covariate and cardiovascular disease, cardiovascular death, and all-cause death. The METS-IR index is positively associated with cardiovascular mortality in the Fine & Gray model analysis (**Supplementary Table 1**). Regardless of gender or different groups of METS-IR index, the METS-IR index is positively correlated with the endpoint events (**Supplementary Table 3**, **Supplementary Figure 3**). When excluding participants who took hypoglycemic and lipid-lowering drugs before baseline, the results remained basically unchanged (**Supplementary Table 4**).

## Discussion

In this prospective study, we explored for the first time the relationship between the METS-IR index and cardiovascular composite endpoints and all-cause mortality in Chinese patients with HTN. The main findings are that METS-IR index, both as a continuous variable and a categorical variable, is positively correlated with cardiovascular composite endpoints and all-cause mortality in Chinese patients with HTN. This correlation is not affected by age, gender, BMI, and current smoking and drinking. Moreover, combining METS-IR parameters with the basic model improved the effectiveness of the basic model in predicting the occurrence of CVD/death.

### Comparison with other studies

There are many alternative methods for estimating IR, including the METS-IR index in this article, as well as quantitative insulin sensitivity test index (QUICKI) and steady-state model evaluation of IR (HOMA-IR) (22, 23). However, the popularization of QUICKI and HOMA-IR in large-scale epidemiology remains challenging due to complex mathematical calculations or the high demands of complex insulin concentration tests. Therefore, in order to better study IR in large-scale epidemiological investigations, researchers have developed some non-insulin-based evaluation indicators that have become predictive factors and biomarkers of insulin resistance. The TyG index, as a simple method to estimate IR, has been shown to have good prognostic value for cardiovascular high-risk population (14, 24). METS-IR, developed by Bello et al, is a new alternative to IR and has a stronger correlation with HEC,compared to other similar indicators (7). However, there is a few researches on the relationship between the METS-IR index and long-term prognosis of hypertension. This is the purpose of this study, which for the first time explores the association between the METS-IR index and cardiovascular composite endpoints and all-cause mortality in Chinese HTN patients.

Hitherto, METS - IR is closely related to a variety of CVD risk factors, such as diabetes, obesity, hypertension, arterial stiffness, hyperuricemia, coronary artery calcification and so on (7, 16, 17, 25). In the CHARLS study, there was a linear positive dose-response relationship between METS-IR and the risk of new-onset cardiovascular disease, stroke, and heart disease among middle-aged and elderly Chinese citizens aged 45 and above, which is consistent with this study (26). In addition, it was found that two studies combined METS-IR parameters with the basic model, significantly improving the effectiveness of the basic model in predicting the occurrence of CVD/death (although the content of the basic model are different). However, no mediating effect of LDL-C was found in our study, possibly due to the fact that the CHARLS study mainly focused on urban populations while our study focused on rural populations. The many differences in lifestyle habits, dietary habits, and other aspects between them may have led to the above situation. The CHARLS study and our study are both prospective cohort studies of the Chinese population, but the sample size included in this study is three times that of the CHARLS study (14220 vs 4540), which may improve the reliability of our results.

In addition, some studies have explored the relationship between IR and all-cause mortality and specific-cause mortality. A prospective cohort study of 6755 Korean adults found that regardless of the presence of IR (HOMA-IR ≥ 2.5), an increasing HOMA-IR over time was associated with a 59%, 87%, 233%, and 367% increased risk of CVD, all-cause mortality, CVD mortality, and MACE mortality (27). Several other studies have shown that higher levels of different IR indicators are independently associated with higher CVD, all-cause and cancer-related mortality rates (28–32). However, other studies have found that the correlation between IR and all-cause and specific mortality rates is not linear (15, 33). For example, Kim et al. found that among 5241 participants aged ≥ 40 years and with normal FBG, high HOMA-IR was associated with higher overall mortality or cardiovascular mortality in lean populations, while in obese populations, high HOMA-IR was associated with lower overall mortality or cardiovascular mortality (34). There are many reasons for the differences, first of all, this may be because we used the METS-IR index instead of the HOMA-IR index to evaluate IR. And we are studying Chinese hypertensive patients rather than Americans. Secondly, from the characteristics of the population, it can be seen that Kim’s study population is overweight, with an average BMI of 26.6, which belongs to the overweight range, while our study population is lean, with an average BMI of 23.6, which belongs to the normal range. Interestingly, it can be seen that the BMI of the dead population in our study is smaller than that of the surviving population, and this difference is significant (**Supplementary Table 2**). A systematic review that the correlation between overweight and obese participants and all-cause mortality compared to normal weight participants in the general population stated that the HRs for all-cause mortality in overweight participants was 0.94 (95% CI 0.91-0.96), while in obese participants it was 1.18 (95% CI 1.12-1.25). Such results not only appear in one study (35). The reasons for such results may be that patients with heavier weight probably experience earlier occurrence of physical discomfort symptoms, and are more likely to receive optimal medication treatment, and the metabolic protective effect of increased body fat and benefits of higher metabolic reserves. Another possibility is that weight loss in high-risk cardiovascular populations may be due to the effects of certain serious diseases, such as tumors, infections, etc.

### Pathogenic mechanism of IR

Although the exact mechanism by which hypertension causes IR is not yet clear, previous studies have shown that IR is an independent predictor of CVD. In clinical practice, hypertension, obesity, and CVD often coexist and promote each other, with IR as the core, accompanied by numerous cardiovascular function and structural abnormalities such as endothelial cell dysfunction (36, 37). The IR we usually refer to is a metabolic disorder caused by damage to the insulin signaling pathway in traditional metabolic tissues (liver, skeletal muscle, and adipose tissue) (38). In fact, the IR not only occurs in some traditional insulin metabolism tissues, but also in cardiovascular, immune cells, and various other tissues. We often refer to the state of impaired insulin signaling pathway and diastolic function of blood vessels as vascular insulin resistance (39, 40). The effect of insulin on the cardiovascular system is closely related to the concentration of insulin in the blood circulation and pathological and physiological conditions. In physiological conditions, insulin exerts a protective effect on the cardiovascular system by stimulating nitric oxide(NO) production; In pathological conditions, hyperinsulinemia may cause vascular damage (40). When vascular insulin resistance occurs, PI3K/NO pathway activated by the insulin is selectively impaired, and compensatory hyperinsulinemia activates MAPK pathway, which leads to vascular smooth muscle cell proliferation, vascular hypertrophy, and promotes the occurrence of hypertension and cardiovascular diseases (39, 40). Some studies speculate that due to the important impact of the insulin PI3K/NO pathway on regulating metabolism and vascular function in vascular endothelial cells, vascular insulin resistance may be an important mechanism by which hypertension increases cardiovascular complications and metabolic diseases (41). Many studies have also confirmed that the degree of insulin resistance is also positively related to atherosclerosis, which is the main risk factor of atherosclerosis (42). The pathogenesis may be that IR induces endothelial dysfunction and glucose metabolism imbalance, and the damaged degradation of apolipoprotein B, leading to abnormal blood lipids and lipid triad (high triglyceride, low high-density lipoprotein cholesterol and high low-density lipoprotein cholesterol), thus promoting the formation of atherosclerotic plaque. The animal model of IR and heart failure also shows that the activation and conduction changes of insulin signaling pathways in myocardial cells occur during heart failure. These changes lead to extracellular matrix protein aggregation, myocardial fibrosis, myocardial hypertrophy, cell apoptosis, decreased calcium processing ability of myocardial cells, abnormal myocardial function, and promote myocardial remodeling, leading to left ventricular remodeling and mitochondrial dysfunction. These pathways are associated with heart failure or diseases related to systemic IR, leading to increased stress exhaustion (43).

In conclusion, pertinent studies have demonstrated that the influence of IR on CVD, including hypertension, coronary heart disease, and heart failure, is facilitated by diverse physiological and biochemical mechanisms. This exacerbates and expedites the advancement of CVD, thereby contributing significantly to the unfavorable prognosis observed in hypertensive patients. This also reminds us of the need for randomized controlled trials to test the impact of interventions targeting insulin resistance on cardiovascular outcomes and mortality in hypertensive patients in future. Similarly, it also means that in clinical practice, clinical doctors should do a good job in managing blood lipids and blood sugar in hypertensive patients to avoid adverse factors such as IR. While previous investigations primarily concentrated on foreign population, the present study addresses the gap by the Chinese population with hypertension. Moreover, this study involves a new indicator of insulin resistance, which has the advantages of convenient use and low cost, and can be extended to large-scale clinical studies or epidemiological investigations for more research on IR. Therefore, more studies are necessary to further explore the underlying mechanisms of association between METS-IR and mortality.

### Advantages and limitations

The advantages of this study include its cohort study design, large sample size, complete follow-up data, adjustment for multiple CVD risk factors, reliable measurement of clinical parameters and anthropometric data, and accurate diagnosis of death information. All of these have added credibility and authenticity to our research results. We also obtained consistent results in subgroup analysis, which enhanced the robustness of our main findings. The consistency results of multiple sensitivity analyses also demonstrate the robustness of the results of this study.

Even so, there are still some limitations to this study. Firstly, this was a substudy of the H-type hypertension. The METS-IR and CVD function of the study participants was only assessed at baseline and the exit visit. More frequent METS-IR and CVD function assays would allow for a more accurate assessment of change in IR and CVD over time. Secondly, although there is some evidences to suggest that METS-IR can serve as an alternative biomarker for evaluating IR, we did not compare the effects of other IR biomarkers on mortality in the same population. However, previous studies have shown a stronger correlation between METS-IR index and HEC compared to other non-insulin IR indices. Thirdly, even if we adjust for the most important known risk factors for CVD, there may still be residual confounding factors such as lifestyle factors, genetic factors, environmental factors, etc. Finally, the participants included in this study were Chinese hypertensive adults; thus, the applicability of our findings to people from other countries remains to be verified.

## Conclusion

In summary, this large, prospective cohort study demonstrated that the METS-IR index, a new IR evaluation index, were independently associated with a higher risk of the cardiovascular composite endpoint and all-cause mortality among Chinese hypertensive population. Importantly, our finding provides an independent indicator for evaluating the prognosis of hypertensive patients.

## Data availability statement

The data analyzed in this study is subject to the following licenses/restrictions: If you need relevant information about the original data, please send an email to the corresponding author of this article for approval. Requests to access these datasets should be directed to HB, huihui_bao77@126.com.

## Ethics statement

The studies involving humans were approved by the Ethics Committee of the Biomedical Research Institute of Anhui Medical University. The studies were conducted in accordance with the local legislation and institutional requirements. The participants provided their written informed consent to participate in this study. Written informed consent was obtained from the individual(s) for the publication of any potentially identifiable images or data included in this article.

## Author contributions

LZ: Writing – original draft. CY: Methodology, Writing – review & editing. TW: Data curation, Methodology, Software, Writing – review & editing. WZ: Data curation, Software, Writing – review & editing. HB: Writing – review & editing. XC: Writing – review & editing.

## References

[1] Hu S-S. Report on cardiovascular health and diseases in China 2021: an updated summary. J Geriatric Cardiol (2023) 20:399–430. doi: 10.26599/1671-5411.2023.06.001 PMC10320777 37416519

[2] Bonora E Kiechl S Willeit J Oberhollenzer F Egger G Targher G. Prevalence of insulin resistance in metabolic disorders: the Bruneck Study. Diabetes (1998) 47:1643–9. doi: 10.2337/diabetes.47.10.1643 9753305

[3] Han K-Y Gu J Wang Z Liu J Zou S Yang C-X. Association between METS-IR and prehypertension or hypertension among Normoglycemia Subjects in Japan: a retrospective study. Front Endocrinol (2022) 13:851338. doi: 10.3389/fendo.2022.851338 PMC8971288 35370984

[4] Ormazabal V Nair S Elfeky O Aguayo C Salomon C Zuñiga FA. Association between insulin resistance and the development of cardiovascular disease. Cardiovasc Diabetol (2018) 17:122. doi: 10.1186/s12933-018-0762-4 30170598 PMC6119242

[5] Bonora E Kiechl S Willeit J Oberhollenzer F Egger G Meigs JB. Insulin resistance as estimated by homeostasis model assessment predicts incident symptomatic cardiovascular disease in caucasian subjects from the general population: the Bruneck study. Diabetes Care (2007) 30:318–24. doi: 10.2337/dc06-0919 17259501

[6] Bornfeldt KE Tabas I. Insulin resistance, hyperglycemia, and atherosclerosis. Cell Metab (2011) 14:575–85. doi: 10.1016/j.cmet.2011.07.015 PMC3217209 22055501

[7] Bello-Chavolla OY Almeda-Valdes P Gomez-Velasco D Viveros-Ruiz T Cruz-Bautista I Romo-Romo A. METS-IR, a novel score to evaluate insulin sensitivity, is predictive of visceral adiposity and incident type 2 diabetes. Eur J Endocrinol (2018) 178:533–44. doi: 10.1530/EJE-17-0883 29535168

[8] Zhang Z Zhao L Lu Y Meng X Zhou X. Association between non-insulin-based insulin resistance indices and cardiovascular events in patients undergoing percutaneous coronary intervention: a retrospective study. Cardiovasc Diabetol (2023) 22:161. doi: 10.1186/s12933-023-01898-1 37386494 PMC10311786

[9] Sato F Nakamura Y Kayaba K Ishikawa S. TG/HDL-C ratio as a predictor of stroke in the population with healthy BMI: The Jichi Medical School Cohort Study. Nutr Metab Cardiovasc Dis (2022) 32:1872–9. doi: 10.1016/j.numecd.2022.05.002 35753859

[10] Mirshafiei H Darroudi S Ghayour-Mobarhan M Esmaeili H AkbariRad M Mouhebati M. Altered triglyceride glucose index and fasted serum triglyceride high-density lipoprotein cholesterol ratio predict incidence of cardiovascular disease in the Mashhad cohort study. Biofactors (2022) 48:643–50. doi: 10.1002/biof.1816 35044705

[11] Chen Y Chang Z Liu Y Zhao Y Fu J Zhang Y. Triglyceride to high-density lipoprotein cholesterol ratio and cardiovascular events in the general population: a systematic review and meta-analysis of cohort studies. Nutr Metab Cardiovasc Dis (2022) 32:318–29. doi: 10.1016/j.numecd.2021.11.005 34953633

[12] Guo J Ji Z Carvalho A Qian L Ji J Jiang Y. The triglycerides-glucose index and the triglycerides to high-density lipoprotein cholesterol ratio are both effective predictors of in-hospital death in non-diabetic patients with AMI. PeerJ (2022) 10:e14346. doi: 10.7717/peerj.14346 36438585 PMC9686411

[13] Wang L Cong H Zhang J Hu Y Wei A Zhang Y. Predictive value of the triglyceride to high-density lipoprotein cholesterol ratio for all-cause mortality and cardiovascular death in diabetic patients with coronary artery disease treated with statins. Front Cardiovasc Med (2021) 8:718604. doi: 10.3389/fcvm.2021.718604 34368266 PMC8333610

[14] Zhu Y Liu K Chen M Liu Y Gao A Hu C. Triglyceride-glucose index is associated with in-stent restenosis in patients with acute coronary syndrome after percutaneous coronary intervention with drug-eluting stents. Cardiovasc Diabetol (2021) 20:137. doi: 10.1186/s12933-021-01332-4 34238294 PMC8268452

[15] Liu X He G Lo K Huang Y Feng Y. The triglyceride-glucose index, an insulin resistance marker, was non-linear associated with all-cause and cardiovascular mortality in the general population. Front Cardiovasc Med (2021) 7:628109. doi: 10.3389/fcvm.2020.628109 33521071 PMC7840600

[16] Wang Z Hui X Huang X Li J Liu N. Relationship between a novel non-insulin-based metabolic score for insulin resistance (METS-IR) and coronary artery calcification. BMC Endocr Disord (2022) 22:274. doi: 10.1186/s12902-022-01180-7 36357872 PMC9647937

[17] Bello-Chavolla OY Antonio-Villa NE Vargas-Vázquez A Martagón AJ Mehta R Arellano-Campos O. Prediction of incident hypertension and arterial stiffness using the non-insulin-based metabolic score for insulin resistance (METS-IR) index. J Clin Hypertens (Greenwich) (2019) 21:1063–70. doi: 10.1111/jch.13614 PMC8030285 31318156

[18] Kim T Kang J. Relationship between obstructive sleep apnea, insulin resistance, and metabolic syndrome: a nationwide population-based survey. Endocr J (2023) 70:107–19. doi: 10.1507/endocrj.EJ22-0280 36171092

[19] Wang Z Li W Li J Liu N. The nonlinear correlation between a novel metabolic score for insulin resistance and subclinical myocardial injury in the general population. Front Endocrinol (Lausanne) (2022) 13:889379. doi: 10.3389/fendo.2022.889379 35685209 PMC9171429

[20] Liu XZ Fan J Pan SJ. METS-IR, a novel simple insulin resistance indexes, is associated with hypertension in normal-weight Chinese adults. J Clin Hypertens (Greenwich) (2019) 21:1075–81. doi: 10.1111/jch.13591 PMC8030630 31282098

[21] Liu H Zha X Ding C Hu L Li M Yu Y. AST/ALT ratio and peripheral artery disease in a Chinese hypertensive population: a cross-sectional study. Angiology (2021) 72:916–22. doi: 10.1177/00033197211004410 33779311

[22] Matthews DR Hosker JP Rudenski AS Naylor BA Treacher DF Turner RC. Homeostasis model assessment: insulin resistance and beta-cell function from fasting plasma glucose and insulin concentrations in man. Diabetologia (1985) 28:412–9. doi: 10.1007/BF00280883 3899825

[23] Brun J-F Ghanassia E Fédou C Bordenave S Raynaud de Mauverger E Mercier J. Assessment of insulin sensitivity (S I) and glucose effectiveness (S G) from a standardized hyperglucidic breakfast test in type 2 diabetics exhibiting various levels of insulin resistance. Acta Diabetol (2013) 50:143–53. doi: 10.1007/s00592-010-0232-2 20981457

[24] Jiao Y Su Y Shen J Hou X Li Y Wang J. Evaluation of the long-term prognostic ability of triglyceride-glucose index for elderly acute coronary syndrome patients: a cohort study. Cardiovasc Diabetol (2022) 21:3. doi: 10.1186/s12933-021-01443-y 34991602 PMC8740408

[25] Fan J Gao ST Wang LJ Qian ZL Zhou ZQ Liu XZ. Association of three simple insulin resistance indexes with prehypertension in normoglycemic subjects. Metab Syndr Relat Disord (2019) 17:374–9. doi: 10.1089/met.2019.0029 31211636

[26] Qian T Sheng X Shen P Fang Y Deng Y Zou G. Mets-IR as a predictor of cardiovascular events in the middle-aged and elderly population and mediator role of blood lipids. Front Endocrinol (2023) 14:1224967. doi: 10.3389/fendo.2023.1224967 PMC10393118 37534205

[27] Lee J-H Jeon S Joung B Lee HS Kwon Y-J. Associations of homeostatic model assessment for insulin resistance trajectories with cardiovascular disease incidence and mortality. ATVB (2023) 43:1719–28. doi: 10.1161/ATVBAHA.123.319200 37470180

[28] Perseghin G Calori G Lattuada G Ragogna F Dugnani E Garancini MP. Insulin resistance/hyperinsulinemia and cancer mortality: the Cremona study at the 15th year of follow-up. Acta Diabetol (2012) 49:421–8. doi: 10.1007/s00592-011-0361-2 22215126

[29] Wu Z Liu L Wang W Cui H Zhang Y Xu J. Triglyceride-glucose index in the prediction of adverse cardiovascular events in patients with premature coronary artery disease: a retrospective cohort study. Cardiovasc Diabetol (2022) 21:142. doi: 10.1186/s12933-022-01576-8 35906587 PMC9338459

[30] Zhou Y Wang C Che H Cheng L Zhu D Rao C. Association between the triglycerideglucose index and the risk of mortality among patients with chronic heart failure: results from a retrospective cohort study in China. Cardiovasc Diabetol (2023) 22:171. doi: 10.1186/s12933-023-01895-4 37420232 PMC10329381

[31] Zhang X Liu F Li W Zhang J Zhang T Yu X. Metabolic score for insulin resistance (METS-IR) predicts adverse cardiovascular events in patients with type 2 diabetes and ischemic cardiomyopathy. DMSO (2023) Volume 16:1283–95. doi: 10.2147/DMSO.S404878 PMC10167964 37179787

[32] Zhang S Wu Z Zhuang Y Sun X Wang J Chen S. The metabolic score for insulin resistance in the prediction of major adverse cardiovascular events in patients after coronary artery bypass surgery: a multicenter retrospective cohort study. Diabetol Metab Syndr (2023) 15:157. doi: 10.1186/s13098-023-01133-7 37461067 PMC10351175

[33] Zhou D Liu X Kenneth L Huang Y Feng Y. A non-Linear association of triglyceride glycemic index with cardiovascular and all-cause mortality among patients with hypertension. Front Cardiovasc Med (2022) 8:778038. doi: 10.3389/fcvm.2021.778038 35155598 PMC8828937

[34] Kim K-S Lee Y-M Lee I-K Kim D-J Jacobs DR Lee D-H. Paradoxical associations of insulin resistance with Total and Cardiovascular Mortality in Humans. Journals Gerontology: Ser A (2015) 70:847–53. doi: 10.1093/gerona/glu194 25326285

[35] McGee DL. Body mass index and mortality: a meta-analysis based on person-level data from twenty-six observational studies. Ann Epidemiol (2005) 15:87–97. doi: 10.1016/j.annepidem.2004.05.012 15652713

[36] Pi X Xie L Patterson C. Emerging roles of vascular endothelium in metabolic homeostasis. Circ Res (2018) 123:477–94. doi: 10.1161/CIRCRESAHA.118.313237 PMC6205216 30355249

[37] Kim J Montagnani M Koh KK Quon MJ. Reciprocal relationships between insulin resistance and endothelial dysfunction: molecular and pathophysiological mechanisms. Circulation (2006) 113:1888–904. doi: 10.1161/CIRCULATIONAHA.105.563213 16618833

[38] Kahn CR Wang G Lee KY. Altered adipose tissue and adipocyte function in the pathogenesis of metabolic syndrome. J Clin Invest (2019) 129:3990–4000. doi: 10.1172/JCI129187 31573548 PMC6763230

[39] Schulman IH Zhou M-S. Vascular insulin resistance: a potential link between cardiovascular and metabolic diseases. Curr Hypertens Rep (2009) 11:48–55. doi: 10.1007/s11906-009-0010-0 19146801

[40] King GL Park K Li Q. Selective Insulin resistance and the development of cardiovascular diseases in diabetes: The 2015 Edwin Bierman Award lecture. Diabetes (2016) 65:1462–71. doi: 10.2337/db16-0152 PMC4878431 27222390

[41] Rask-Madsen C Kahn CR. Tissue-specific insulin signaling, metabolic syndrome, and cardiovascular disease. Arterioscler Thromb Vasc Biol (2012) 32:2052–9. doi: 10.1161/ATVBAHA.111.241919 PMC3511859 22895666

[42] Abraham WT. Preventing cardiovascular events in patients with diabetes mellitus. Am J Med (2004) 116 Suppl:5A:39S–46S. doi: 10.1016/j.amjmed.2003.10.019 15019862

[43] Velez M Kohli S Sabbah HN. Animal models of insulin resistance and heart failure. Heart Fail Rev (2014) 19:1–13. doi: 10.1007/s10741-013-9387-6 23456447 PMC3688662

