# Supplementary material for: Association of the metabolic score for insulin resistance with cardiovascular diseases, cardiovascular and all-cause mortality in Chinese hypertensive population
Source: Front Endocrinol (Lausanne). 2024 Mar 8;14:1326436. doi: 10.3389/fendo.2023.1326436 (PMC10957551; doi:10.3389/fendo.2023.1326436)
Supplement: Supplementary file 1 [file DataSheet_1.pdf]

Association of the metabolic score for insulin resistance (METS-IR) with cardiovascular diseases, cardiovascular and all-cause mortality in Chinese hypertensive population

Liting Zhang[1,2,3,4], Hongdong Jiang[5], Chao Yu[1,2,3,4], Tao Wang[1,2,3,4], Wei Zhou[1,2,3,4], Linjuan Zhu[1,2,3,4], Jine Liu[1,2,3,4], Dan Sun[1,2,3,4], Chuanli Yu[1,2,3,4], Huihui Bao[*1,2,3,4], Xiaoshu Cheng[1,2,3,4]

1. Department of Cardiovascular Medicine, the Second Affiliated Hospital of Nanchang University, Nanchang of Jiangxi, China.
2. Center for Prevention and Treatment of Cardiovascular Diseases, the Second Affiliated Hospital of Nanchang University, Nanchang of Jiangxi, China.
3. Jiangxi Provincial Cardiovascular Disease Clinical Medical Research Center, Nanchang of Jiangxi, China.
4. Jiangxi Sub-center of National Clinical Research Center for Cardiovascular Diseases, Nanchang of Jiangxi, China.
5. General Department of Jiangwan Town Central Health Center, Wuyuan County, Jiangxi, China.

[*]**Correspondence:**

Huihui Bao
Huihui_bao77@126.com

Figure S1：Research population selection process diagram.

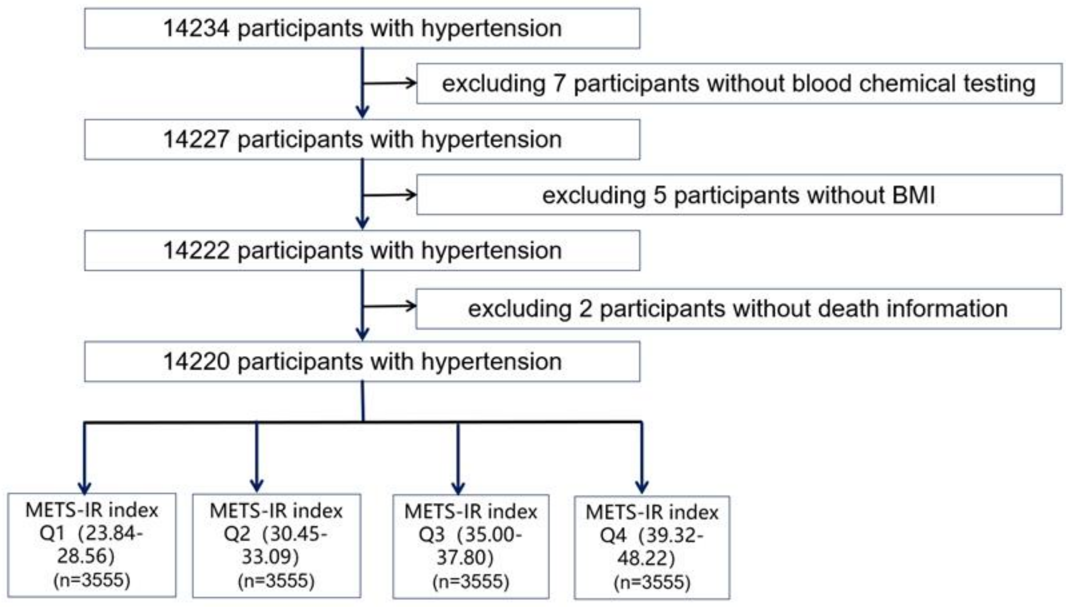

Figure S2：Analysis of the Schoenfeld residuals to assess the proportional hazards assumption.

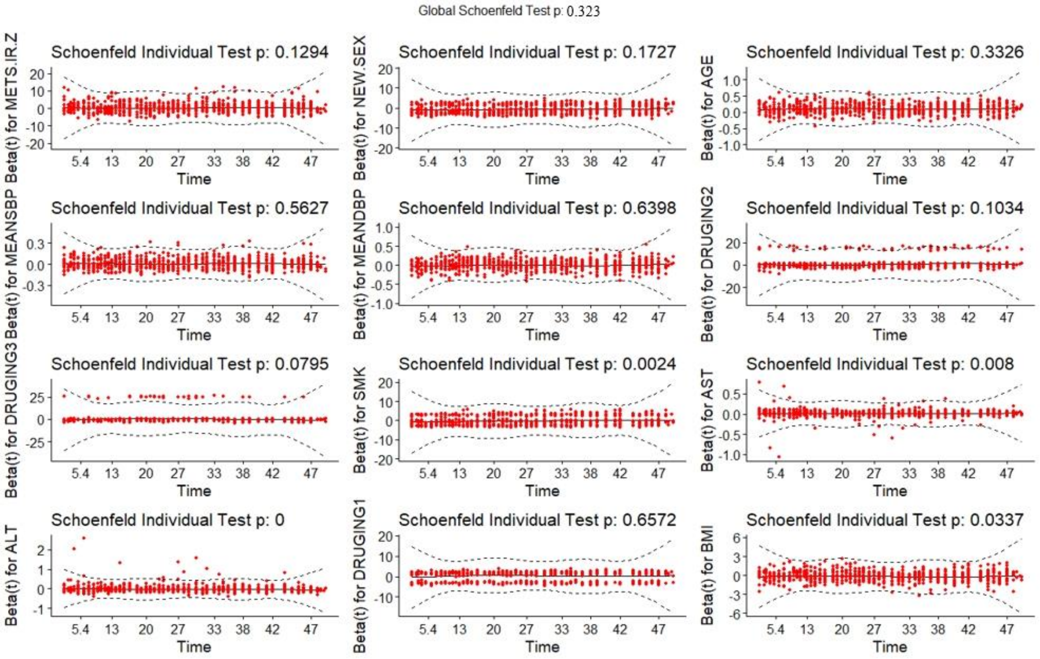

Figure S3: Restricted cubic spline regression analysis of METS-IR index with CVD(A), cardiovascular death(B), all-cause death(C) by gender stratification.

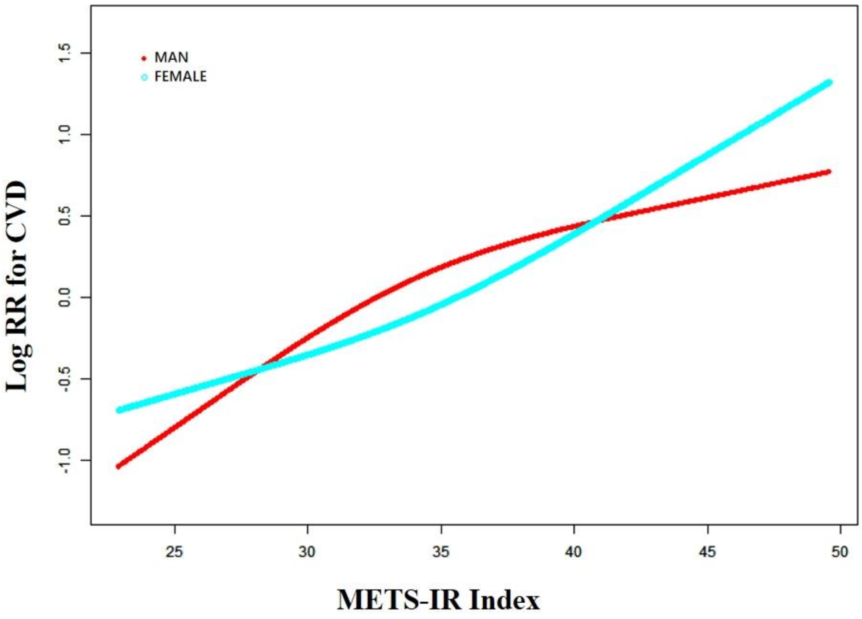

A.

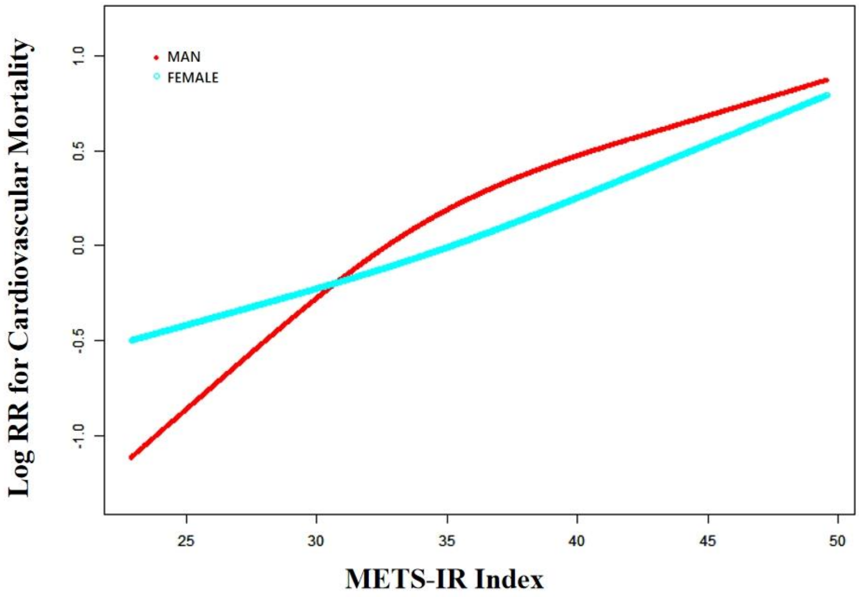

B.

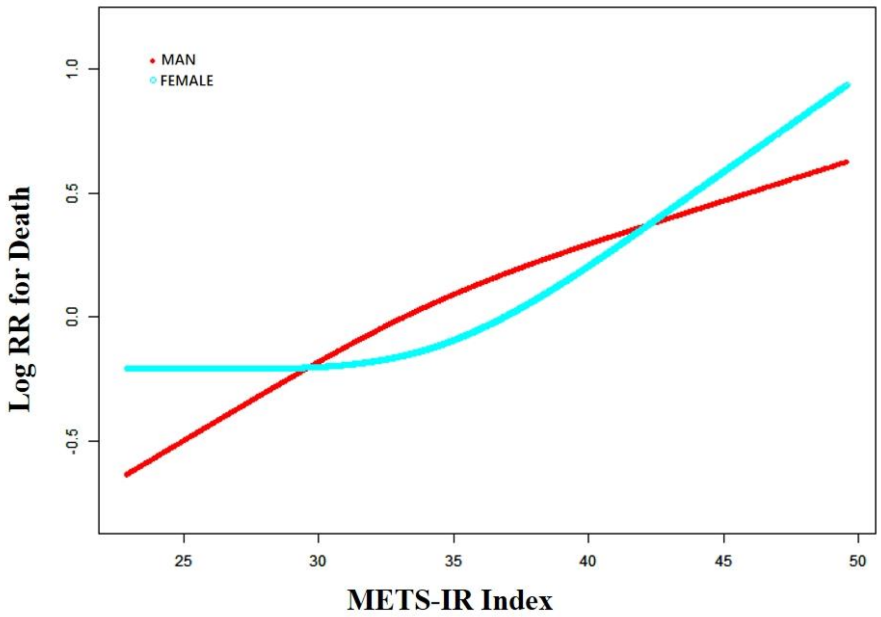
MAN
FEMALE
Log RR for Death
1.0
0.5
0.0
-0.5
25
30
35
40
45
50
METS-IR Index

C.
