# Supplementary material for: Association of the metabolic score for insulin resistance with cardiovascular diseases, cardiovascular and all-cause mortality in Chinese hypertensive population
Source: Front Endocrinol (Lausanne). 2024 Mar 8;14:1326436. doi: 10.3389/fendo.2023.1326436 (PMC10957551; doi:10.3389/fendo.2023.1326436)
Supplement: Supplementary file 2 [file Table_1.pdf]

Association of the metabolic score for insulin resistance (METS-IR) with cardiovascular diseases, cardiovascular and all-cause mortality in Chinese hypertensive population

Liting Zhang[1,2,3,4], Hongdong Jiang[5], Chao Yu[1,2,3,4], Tao Wang[1,2,3,4], Wei Zhou[1,2,3,4], Linjuan Zhu[1,2,3,4], Jine Liu[1,2,3,4], Dan Sun[1,2,3,4], Chuanli Yu[1,2,3,4], Huihui Bao[*1,2,3,4], Xiaoshu Cheng[1,2,3,4]

1. Department of Cardiovascular Medicine, the Second Affiliated Hospital of Nanchang University, Nanchang of Jiangxi, China.
2. Center for Prevention and Treatment of Cardiovascular Diseases, the Second Affiliated Hospital of Nanchang University, Nanchang of Jiangxi, China.
3. Jiangxi Provincial Cardiovascular Disease Clinical Medical Research Center, Nanchang of Jiangxi, China.
4. Jiangxi Sub-center of National Clinical Research Center for Cardiovascular Diseases, Nanchang of Jiangxi, China.
5. General Department of Jiangwan Town Central Health Center, Wuyuan County, Jiangxi, China.

[*]Correspondence:

Huihui Bao
Huihui_bao77@126.com

Table S1: The relationship between METS-IR index and cardiovascular mortality in the Fine and Gray competing risk model.

Variables	Model 1[*]	
	HR/95%CI	*P* value
METS-IR Per SD	1.56(1.07,2.29)	0.021

Model 1[*] adjusted for age, sex, BMI, current smoking status, mean SBP, mean DBP, hypoglycemic drugs, lipid-lowering drugs, antihypertensive drugs, AST, ALT.
METS-IR: metabolic score for insulin resistance; BMI: body mass index; SBP: systolic blood pressure; DBP: diastolic blood pressure; AST: aspartate transaminase; ALT: alanine transaminase.

Table S2: Baseline characteristics of the study population according to their survival status.

Variables	Total	Survival	Death	*P* value
**Sex, n (%)**				<0.001
Male	6714(47.2%)	6209(46.3%)	505(62.7%)	
Female	7506(52.8%)	7206(53.7%)	300(37.3%)	
Age, year	63.8 ±9.4	63.3 ±9.2	72.6 ±8.4	
Height, cm	156.1 ±8.2	156.1 ±8.2	155.0 ±8.4	<0.001
Weight, cm	57.7 ±10.7	58.0 ±10.7	52.4 ±10.0	<0.001
BMI, kg/m2	23.6 ±3.7	23.7 ±3.7	21.8 ±3.5	<0.001
SBP, mmHg	148.4 ±17.9	148.2 ±17.6	150.8 ±21.6	<0.001
DBP, mmHg	88.9 ±10.8	89.1 ±10.6	85.2 ±12.1	<0.001
HR, bmp	76.7 ±14.2	76.5 ±13.9	79.7 ±17.5	<0.001
Current smoking	3658(25.7%)	3413(25.4%)	245(30.4%)	0.002
Current drinking	3062(21.5%)	2910(21.7%)	152(18.9%)	0.059
Use of hypoglycemic drugs	754(5.3%)	697(5.2%)	57(7.1%)	0.394
Use of lipid-lowering drugs	506(3.6%)	473(3.5%)	33(4.1%)	0.020
Use of antihypertensive drugs	9219(64.8%)	8649(64.5%)	570(70.8%)	<0.001
**Education**				<0.001
Primary and below	9207(79.9%)	8585(79.4%)	622(88.9%)	
Secondary school	2135(18.5%)	2063(19.1%)	72(10.3%)	
University and above	176(1.5%)	170(1.6%)	6(0.9%)	
**Life level**				0.036
Rich	1503(13.0%)	1403(13.0%)	100(14.3%)	
Ordinary	7741(67.2%)	7301(67.5%)	440(62.9%)	
Poor	2274(19.7%)	2114(19.5%)	160(22.9%)	
**Laboratory indicators**				
FBG, mmol/L	6.2 ±1.6	6.2 ±1.6	6.3 ±2.1	0.076
TG, mmol/L	1.8 ±1.3	1.8 ±1.3	1.5±1.0	<0.001
HDL, mmol/L	1.6 ±0.4	1.6 ±0.4	1.6 ±0.4	0.641
LDL, mmol/L	3.0 ±0.8	3.0 ±0.8	2.8±0.8	<0.001
AST, mmol/L	26.8 ±15.8	26.8 ±15.8	26.8±16.3	0.985
ALT, mmol/L	20.5 ±16.6	20.7±16.7	17.0±14.0	<0.001

BMI: body mass index; SBP: systolic blood pressure; DBP: diastolic blood pressure; HR: heart rate; FBG: fasting blood glucose; TG: triglyceride; HDL: high-density lipoprotein; LDL: low-density lipoprotein; METS-IR: metabolic score for insulin resistance; AST: aspartate transaminase; ALT: alanine transaminase

Table S3： Multivariable-adjust HRs and 95%CI of the METS-IR index quartiles associated with CVD, cardiovascular Mortality, all-cause mortality.

Variables	Event, n (%)	Model 1*	
		HR/95%CI	*P* Value
**CVD**			
METS-IR Per SD		1.66(1.30,2.12)	<0.001
METS-IR Quartiles			
Q1	103(3.62%)	1 [Reference]	
Q2	81(2.85%)	1,34(0.96,1.89)	0.089
Q3	71(2.50%)	1.58(1.04,2.39)	0.031
Q4	59(2.07%)	1.90(1.15,3.13)	0.012
Q5	55(1.93%)	2.67(1.45,4.92)	0.002
P For Trend			0.002
**Cardiovascular Death**			
METS-IR Per SD		1.57(1.22,2.02)	<0.001
METS-IR Quartiles			
Q1	129(4.54%)	1 [Reference]	
Q2	107(3.76%)	1.66(1.22,2.26)	0.001
Q3	64(2.25%)	1.44(0.95,2.18)	0.085
Q4	60(2.11%)	2.03(1.25,3.32)	0.004
Q5	39(1.37%)	2.15(1.15,4.04)	0.017
P For Trend			0.022
**All-cause Death**			
METS-IR Per SD		1.33(1.11,1.60)	0.002
METS-IR Quartiles			
Q1	298(10.48%)	1 [Reference]	
Q2	177(6.22%)	1.10(0.88,1.38)	0.390
Q3	139(4.89%)	1.22(0.92,1.61)	0.173
Q4	104(3.66%)	1.31(0.93,1.84)	0.129
Q5	87(3.06%)	1.70(1.11,2.16)	0.024
P For Trend			0.002

Model 1* adjusted for age, sex, BMI, current smoking status, mean SBP, mean DBP, hypoglycemic drugs, lipid-lowering drugs, antihypertensive drugs, AST, ALT.

METS-IR: metabolic score for insulin resistance; CVD: cardiovascular disease; BMI: body mass index; SBP: systolic blood pressure; DBP: diastolic blood pressure; AST: aspartate transaminase; ALT: alanine transaminase.

Table S4: Multivariate adjusted HR and 95% CI of the METS-IR index quartile associated with CVD, cardiovascular death, and all-cause death in populations who were excluded taking hypoglycemic and lipid-lowering drugs at baseline.

Variables	Event, n (%)	Model 1		Model2	
		HR/95%CI	*P* Value	HR/95%CI	*P* Value
**CVD**					
METS-IR Per SD		1.85(1.43,2.39)	<0.001	1.90(1.46,2.46)	<0.001
METS-IR Quartiles					
Q1	109(3.3%)	1 [Reference]		1 [Reference]	
Q2	83(2.5%)	1.53(1.08,2.16)	0.017	1.53(1.08,2.17)	0.016
Q3	65(2.0%)	1.93(1.23,3.02)	0.004	1.94(1.23,3.05)	0.004
Q4	55(1.7%)	2.96(1.65,5.32)	<0.001	3.06(1.70,5.53)	<0.001
P For Trend			<0.001		<0.001
**Cardiovascular Death**					
METS-IR Per SD		1.63(1.25,2.12)	<0.001	1.74(1.34,2.27)	<0.001
METS-IR Quartiles					
Q1	147(4.5%)	1 [Reference]		1 [Reference]	
Q2	94(2.9%)	1.30(0.94,1.79)	0.111	1.37(0.99,1.88)	0.058
Q3	72(2.2%)	1.57(1.03,2.40)	0.038	1.68(1.09,2.57)	0.018
Q4	41(1.3%)	1.60(0.89,2.88)	0.115	1.79(0.99,3.23)	0.053
P For Trend			0.063		0.027
**All-cause Death**					
METS-IR Per SD		1.37(1.13,1.65)	0.001	1.45(1.20,1.75)	<0.001

METS-IR Quartiles					
Q1	322(9.9%)	1 [Reference]		1 [Reference]	
Q2	180(5.5%)	1.12(0.89,1.40)	0.336	1.15(0.92,1.44)	0.222
Q3	135(4.1%)	1.31(0.97,1.76)	0.078	1.37(1.01,1.84)	0.041
Q4	84(2.6%)	1.44(0.96,2.16)	0.081	1.56(1.03,2.34)	0.034
P For Trend			0.055		0.022

Model 1 adjusted for age, sex, BMI

Model 2 adjusted for age, sex, BMI, current smoking status, mean SBP, mean DBP, AST, ALT

METS-IR: metabolic score for insulin resistance; CVD: cardiovascular disease; BMI: body mass index; SBP: systolic blood pressure; DBP: diastolic blood pressure; AST: aspartate transaminase; ALT: alanine transaminase.

Table S5: Incremental predictive value of METS- IR index.

	C-Statistic(95%CI)	Continuous NRI	*P*-value	IDI	*P*-value
**CVD**					
Basic model	0,754(0.729,0.779)	1 [Reference]		1 [Reference]	
Basic model +METS-IR	0.762(0.737,0.787)	0.128	**0.027**	0.002	0.093
**Cardiovascular Mortality**					
Basic model	0.805(0.785,0.826)	1 [Reference]		1 [Reference]	
Basic model +METS-IR	0.808(0.787,0.828)	0.091	**0.047**	0.002	**0.027**
**All-cause Mortality**					
Basic model	0.784(0.769,0.799)	1 [Reference]		1 [Reference]	
Basic model +METS-IR	0.786(0.711,0.801)	0.041	0.113	0.001	**0.040**

Basic model included age, sex, BMI, SBP, DBP, smoking, drinking.

METS- IR: Metabolic score for insulin resistance; CVD: Cardiovascular disease; NRI: Net Reclassification Improvement; IDI: Integrated Discrimination Improvement; BMI: body mass index.
